# LungINFseg: Segmenting COVID-19 Infected Regions in Lung CT Images Based on a Receptive-Field-Aware Deep Learning Framework

**DOI:** 10.3390/diagnostics11020158

**Published:** 2021-01-22

**Authors:** Vivek Kumar Singh, Mohamed Abdel-Nasser, Nidhi Pandey, Domenec Puig

**Affiliations:** 1 Department of Computer Engineering and Mathematics, Universitat Rovira i Virgili, 43007 Tarragona, Spain; vivekkr.singh90@gmail.com (V.K.S.); domenec.puig@urv.cat (D.P.); 2 Department of Electrical Engineering, Aswan University, 81542 Aswan, Egypt; 3 Department of Medicine and Health Sciences, Universitat Rovira i Virgili, 43201 Reus, Spain; nidhi.pandey3790@gmail.com

**Keywords:** COVID-19, CT slices, deep learning, image segmentation

## Abstract

COVID-19 is a fast-growing disease all over the world, but facilities in the hospitals are restricted. Due to unavailability of an appropriate vaccine or medicine, early identification of patients suspected to have COVID-19 plays an important role in limiting the extent of disease. Lung computed tomography (CT) imaging is an alternative to the RT-PCR test for diagnosing COVID-19. Manual segmentation of lung CT images is time consuming and has several challenges, such as the high disparities in texture, size, and location of infections. Patchy ground-glass and consolidations, along with pathological changes, limit the accuracy of the existing deep learning-based CT slices segmentation methods. To cope with these issues, in this paper we propose a fully automated and efficient deep learning-based method, called LungINFseg, to segment the COVID-19 infections in lung CT images. Specifically, we propose the receptive-field-aware (RFA) module that can enlarge the receptive field of the segmentation models and increase the learning ability of the model without information loss. RFA includes convolution layers to extract COVID-19 features, dilated convolution consolidated with learnable parallel-group convolution to enlarge the receptive field, frequency domain features obtained by discrete wavelet transform, which also enlarges the receptive field, and an attention mechanism to promote COVID-19-related features. Large receptive fields could help deep learning models to learn contextual information and COVID-19 infection-related features that yield accurate segmentation results. In our experiments, we used a total of 1800+ annotated CT slices to build and test LungINFseg. We also compared LungINFseg with 13 state-of-the-art deep learning-based segmentation methods to demonstrate its effectiveness. LungINFseg achieved a dice score of 80.34% and an intersection-over-union (IoU) score of 68.77%—higher than the ones of the other 13 segmentation methods. Specifically, the dice and IoU scores of LungINFseg were 10% better than those of the popular biomedical segmentation method U-Net.

## 1. Introduction

Coronavirus disease 2019 (COVID-19) is an infectious disease caused by severe acute respiratory syndrome coronavirus 2 (SARS-CoV-2), which is still threatening humans worldwide. The World Health Organization (WHO) declared that COVID-19 (the novel coronavirus disease) is a global pandemic on the 11 March 2020 [1]. Due to unavailability of an appropriate vaccine or medicine, the early diagnosis of COVID-19 disease is very crucial to saving many people’s lives and protecting frontline workers. One of the gold standard COVID-19 detection methods is RT-PCR (reverse transcription-polymerase chain reaction); note that the RT-PCR test is time-consuming and has low sensitivity [2]. Besides, RT-PCR testing capacity is not enough in all countries and the required material is limited in hospitals considering the number of possible infections. It should be noted that chest CT imaging, which is a non-invasive, routine diagnostic tool for pneumonia, has been used to supplement RT-PCR testing to detect COVID-19 [3]. The study of [4] concluded that chest CT images reveal some noted imaging features of COVID-19, including ground-glass opacification (GGO) and consolidative opacities overlaid on GGO, which can be found mainly in the lower lobes. These features can help detect COVID-19 early before noticing the clinical symptoms. Additional features of COVID-19, such as pleural/septal thickening, subpleural involvement, and bronchiectasis, can be noticed in the later stages of the disease. It is worth noting that some related works, such as [5,6], suggest the use of chest radiographs (CXR) due to their widespread availability and portability, non-invasiveness, and faster acquisition and visual analysis. However, CTs have higher accuracy than CXR and allow diminishing the false negative errors from repeated swab analysis [7].

**Figure 1:**
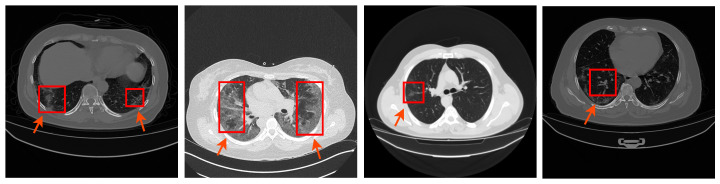
Four examples of COVID-19 existing in chest CT images (COVID-19 infection is highlighted by red boxes).

Considering the massive number of daily infections, radiologists encounter difficulties in visually inspecting all CT images to identify the salient imaging features of COVID-19. Hence, there is a requirement to develop accurate automated tools to segment COVID-19 infection in the chest CT images at the lung level. Figure 1 shows four examples of COVID-19 existing in the chest CT images. Radiologists can notice regions of patchy ground-glass and consolidations in COVID-19 CT images. These artefacts, and pathological changes, can limit the accuracy of automated lobe segmentation methods. Computer-aided diagnosis (CAD) systems can support the radiologist in identifying the COVID-19 infection from lung CT images. Since COVID-19 is a new disease, therefore CAD systems are helpful to instruct less experienced radiologists. The main aim of the CAD systems is to provide a precise segmentation of the infected region from lung CT.

A growing number of research groups across the globe have shown that medical image segmentation algorithms based on deep learning have a tremendous capacity that can help detect and segment COVID-19 infections from lung CT ges. In [8], a deep learning-based method is suggested to segment COVID-19 infection by aggregating residual transformations and employing soft attention techniques to learn significant feature representations from lung CT images. In [9], an encoder–decoder network with feature variation and progressive atrous spatial pyramid pooling blocks is proposed to segment the infected region. A total of 21,658 annotated chest CT images (861 confirmed COVID-19 patients) were used to train the segmentation model. With CT images of 130 patients, a dice score of 72.60% was achieved. The authors of [10] investigated the effectiveness of deep learning models for segmenting pneumonia infected area in CT images for the detection of COVID-19. Specifically, they studied the efficacy of U-Net and a fully convolutional neural network (FCN) with CT images. With a dataset of 10 axial volumetric CT scans of confirmed COVID-19 pneumonia patients, the FCN model achieved an F1-score (dice score) of 57% approximately.

In [11], a COVID-19 pneumonia lesion segmentation network, called COPLE-Net, was proposed to handle the lesions with various scales and appearances. In this model, a noise-robust dice loss (a generalization of dice loss) is introduced. This segmentation model has been trained and evaluated on images of 558 COVID-19 patients collected from 10 different hospitals, achieving a dice score of 80.29%. Fan et al. [12] employed a parallel partial decoder to aggregate features from high-level layers to generate coarse representations. Then, they used recurrent reverse attention and edge attention guidance approaches to model the boundaries of infected areas. In [12], Fan et al. also proposed a semi-supervised segmentation framework, Semi-Inf-Net, based on a randomly selected propagation strategy that needs a few labeled pieces of data for training. The Semi-Inf-Net model achieved a dice score of 59.70% with nine real CT volumes with 638 slices. Muller et al. [13] used different preprocessing methods and on-the-fly data augmentation techniques for training the 3D U-Net architecture using a small CT image dataset. They achieved a dice score of 76.10% with 20 CT volumes.

As mentioned above, patchy ground-glass and consolidations, and pathological changes, limit the accuracy of the existing segmentation methods. Receptive field (field-of-view), a region of neurons in a particular layer that affects a neuron in the next layer, is a vital concept in designing CNN. Large receptive fields could help deep learning models to learn contextual information and COVID-19 infection-related features that yield accurate segmentation results. The most common ways to enlarge the receptive field of a CNN is to increase the depth of the network, use pooling operations, and enlarge of sizes of filters. The increase of network depth or enlargement of sizes of filters significantly increases the computational cost, and pooling operations yield information loss. Dilated convolution [14] is also employed to enlarge the receptive fields of CNNs by inserting zeros in the filters, which has no computational cost.

In an attempt to address the problems stated above, we propose a fully automated and efficient deep learning-based method called LungINFseg, to segment the COVID-19 infection in lung CT images. Specifically, we propose the receptive-field-aware (RFA) module that can enlarge the receptive field of a segmentation models and increase the learning ability of the model without information loss. RFA comprises convolution layers to extract COVID-19 features, dilated convolution consolidated with learnable parallel-group convolution to enlarge the receptive field, frequency domain features obtained by discrete wavelet transform (DWT), which also enlarge the receptive field, and an attention mechanism to promote COVID-19-related features. We compared LungINFseg with 13 state-of-the-art deep learning-based segmentation methods to demonstrate its effectiveness. The main contributions of this article are listed below:

1. We propose a fully automated and efficient deep learning based method to segment the COVID-19 infection in lung CT images.
2. We propose the RFA module that can enlarge the receptive field of the segmentation models and increase the learning ability of the model without information loss.
3. We present a comprehensive comparison with 13 state-of-the-art segmentation models, namely, FCN [15], UNet [16], SegNet [17], FSSNet [18], SQNet [19], ContextNet [20], EDANet [21], CGNet [22], ERFNet [23], ESNet [24], DABNet [25], Inf-Net [12], and MIScnn [26].
4. Extensive experiments were performed to provide ablation studies that add a thorough analysis of the proposed LungINFSeg (e.g., the effect of resolution size and variation of the loss function). To reproduce the results, the source code of the proposed model is publicly available at https://github.com/vivek231/LungINFseg.

This article is structured as follows: Section 2 explains the proposed LungINFseg model. Section 3 presents experimental results with an ablation study about the features of the proposed model. Finally, Section 4 concludes the article.

## 2. Methodology

**Figure 2:**
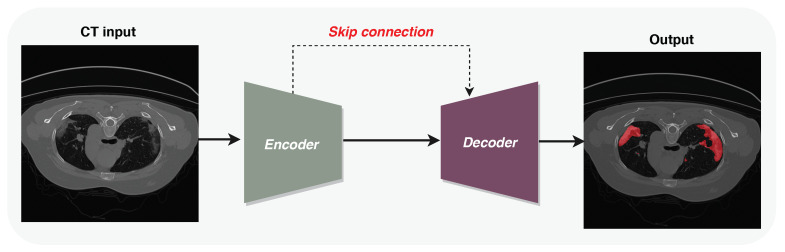
Framework of proposed LungINFseg.

Figure 2 presents the framework of the proposed LungINFseg model, which includes encoder and decoder networks. LungINFseg receives CT images as input and produces binary masks highlighting the infected regions. The features of each encoder block are bypassed to the corresponding decoder block to preserve the spatial feature information. In the following sections, we explain each part in detail.

### 2.1. Encoder

**Figure 3:**
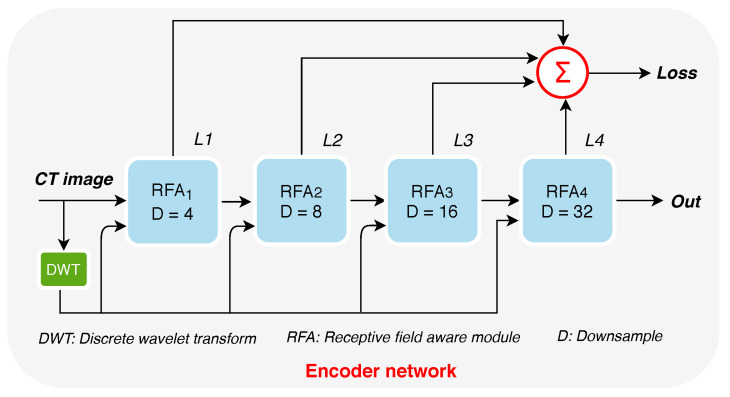
The encoder network. L1,L2,L3,L4 represent the block-wise losses. *D* refers to the down-sampling rate. Out represents the output features generated by the Encoder network. RFA refers to the receptive field aware module. DWT refers to the discrete wavelet transform.

Figure 3 shows the encoder network that comprises four RFA blocks. As one can see, the CT images are fed into the main DWT is to obtain multi-band multi-scale decomposition of input lung CT images. The resulting DWT representations of CT images serve as an inputs to RFA blocks.

#### 2.1.1. Increasing the Receptive Fields Using Discrete Wavelet Transform (DWT)

**Figure 4:**
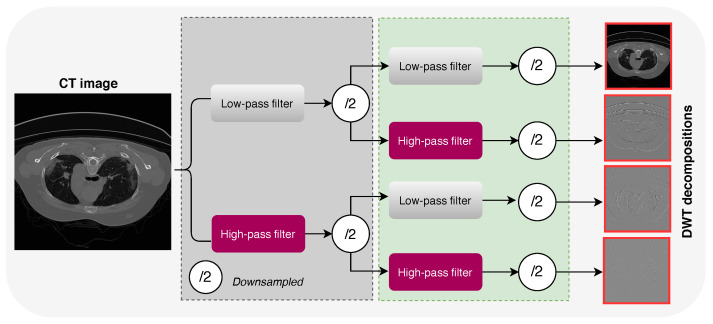
Illustration of decomposing a CT image into sub-band using DWT.

As the human visual system has unequal sensitivity to frequency components, inserting frequency information oto the deep learning-based COVID-19 infection segmentation models can significantly improve their performance. In this study, DWT was utilized to extract COVID-19 infection-relevant contextual information, enlarge the receptive field, and preserve image contextual and spatial information. The use of DWT can enlarge the receptive field of CNNs and also increase the amount of data, which enhances the training process. DWT uses filter banks for recognizing both time and frequency resolutions at the same time [27]. In this work, we use 2D DWT with four Haar filters, namely, $f_{LL}={\left[1\;1;1\;1\right]}^{T}$, $f_{LH}={\left[-1\;-1;1\;1\right]}^{T}$, $f_{HL}={\left[-1\;1;-1\;1\right]}^{T}$, and $f_{HH}={\left[1\;-1;-1\;1\right]}^{T}$, to decompose a particular lung CT image *x* into four sub-bands, i.e., $x_{LL}$, $x_{LH}$, $x_{HL}$, and $x_{HH}$, as shown in Figure 4. The decomposition process can be expressed as follows [28]:

(1) $$\left\{\begin{array}{c} x_{LL}\left(i,j\right)=x\left(2i-1,2j-1\right)+x\left(2i-1,2j\right)+x\left(2i,2j-1\right)+x\left(2i,2j\right)\\ x_{LH}\left(i,j\right)=-x\left(2i-1,2j-1\right)-x\left(2i-1,2j\right)+x\left(2i,2j-1\right)+x\left(2i,2j\right)\\ x_{HL}\left(i,j\right)=-x\left(2i-1,2j-1\right)+x\left(2i-1,2j\right)-x\left(2i,2j-1\right)+x\left(2i,2j\right)\\ x_{HH}\left(i,j\right)=x\left(2i-1,2j-1\right)-x\left(2i-1,2j\right)-x\left(2i,2j-1\right)+x\left(2i,2j\right) \end{array}\right.$$

**Figure 5:**
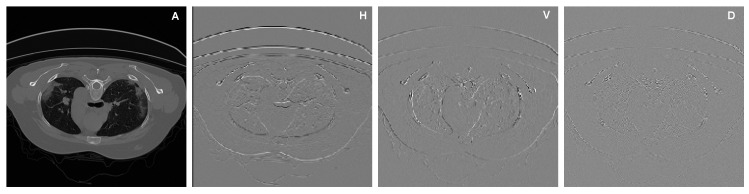
Illustration of a zoom-in visualization of decomposing a CT image into four sub-bands using DWT. A, H, V and D refer to Approximation, Horizontal Detail, Vertical Detail and Diagonal Detail, respectively.

As shown in Figure 4, the input CT is convoluted with low-pass and high-pass filters. While the output of each filter contains half the frequency content, it has the same size as the input CT image. Therefore, the outputs of the low and high branches together comprise the same frequency content as the input CT image; however, the amount of data is doubled, which improves the training process of the proposed model (a kind of data augmentation). Figure 5 shows a zoom-in visualization of the decomposition for a CT image into four sub-bands using DWT. It should be noted that DWT is related to the pooling operation and dilated filtering [29]. Assume that we make an average pooling with a factor 2 on input image *x*; we get $x_{pooling}\left(i,j\right)=\left(x(2i-1,2j-1)+x(2i-1,2j)+x(2i,2j-1)+x(2i,2j)\right)/4$. As one can see in Equation (1), DWT decomposition is connected to the average pooling: for example, the only difference between $x_{LL}$ and $x_{pooling}$ is the fixed coefficient 1/4. In turn, the decomposition of an image into sub-images using DWT is relatively connected to dilated filtering.

#### 2.1.2. Receptive-Field-Aware (RFA) Module

**Figure 6:**
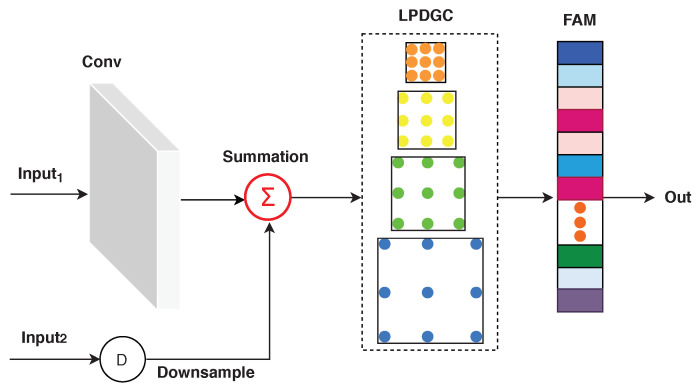
Proposed RFA module. Conv refers to convolution layers. D refers to the down-sampling rate. Out represents the output features generated by the RFA module. LPDGC refers to the learnable parallel dilated group convolutional block. FAM refers to the feature attention module.

Figure 6 shows the structure of the RFA module, which includes convolutional layers obtained from ResNet-18 pre-trained on ImageNet [30], a learnable parallel dilated group convolutional block (LPDGC), and a feature attention module (FAM). RFA encoding layers can learn low-level features from lung CT images, such as spatial information (e.g., shape, edge, intensity, and texture) in the training phase. As one can see in Figure 6, the RFA block receives two inputs. Input${}_{1}$ represents the features extracted in the previous layers (except in the first RFA block, input${}_{1}$ represents the input CT images). Input${}_{2}$ represents the DWT decompositions of the input CT image. Input${}_{1}$ is fed into a convolution layer with a kernel of size 3×3 and a stride of 1. The resulting features are summed with input${}_{2}$ and then fed into LPDGC and FAM modules. Note that the DWT features are resized to the size of Input${}_{1}$ using bilinear interpolation before the summation process.

#### Learnable Parallel Dilated Group Convolutional (LPDGC) Block

**Figure 7:**
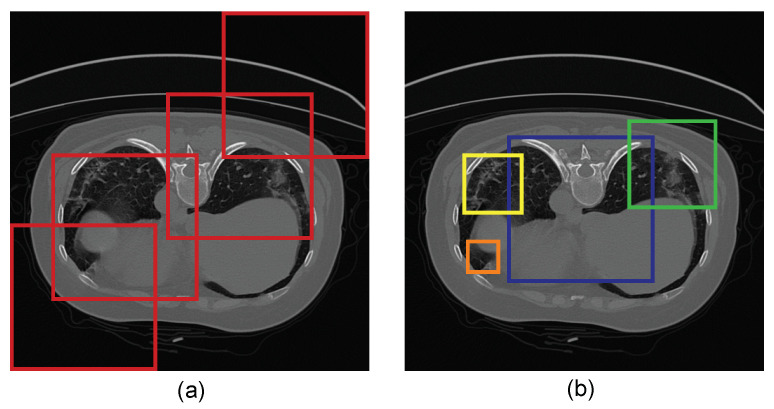
Illustration of receptive fields. (**a**) Receptive fields in the same layer with the same size kernel that capture more background pixels, and (**b**) receptive fields with varying dilation rates (shown in four different colored boxes) which capture the small and relevant regions.

**Figure 8:**
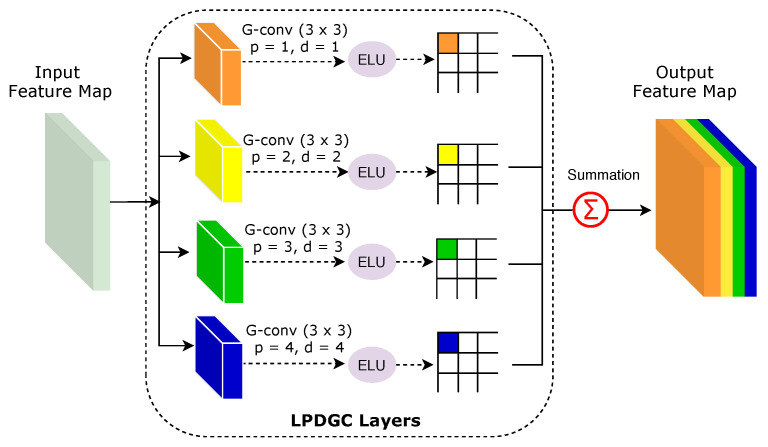
Illustration of the LPDGC block. Here, p and d refer to the padding and dilation rates respectively. ELU refers to the exponential linear unit activation function.

Figure 7 illustrates how receptive fields with varying dilation rates can capture the small and relevant regions in CT images. In this work, we propose the use of the LPDGC block, in which the conventional convolutional filters employed in the parallel dilated group convolutional (PDGC) block are replaced by a fully learnable group convolution mechanism [31]. Figure 8 shows the architecture of the LPDGC block, which comprises four group convolution (G-conv) layers with different dilation rates (1, 2, 3, and 4) followed by an exponential linear unit (ELU) activation function. The kernel size of each G-conv layer is 3×3.

The main goal of learnable group convolution methods is to design a dynamic and efficient mechanism for group convolution, in which input channels and filters in each group are learned during the training phase. In general, the grouping structure can be expressed as two binary selection matrices for channels ($S^{k}$) and filters ($T^{k}$), as follows:

(2) $$S^{k}={\left[\begin{array}{cccc} s_{11} & s_{12} & \cdots & s_{1G}\\ s_{21} & s_{22} & \cdots & s_{2G}\\ \vdots & \vdots & \ddots & \vdots \\ s_{C1} & s_{C2} & \cdots & s_{CG} \end{array}\right]}_{C\times G}$$

(3) $$T^{k}={\left[\begin{array}{cccc} t_{11} & t_{12} & \cdots & t_{1G}\\ t_{21} & t_{22} & \cdots & t_{2G}\\ \vdots & \vdots & \ddots & \vdots \\ t_{N1} & t_{N2} & \cdots & t_{NG} \end{array}\right]}_{N\times G}$$

The size of $S^{k}$ is C×G, and the size of $T^{k}$ is N×G, where, C, N, and G refer to the numbers of channels, filters, and groups respectively. It should be noted that the elements of $S^{k}$ and $T^{k}$ are set as 1 or 0 during the training process, where $s^{k}$(*i*, *j*) = 1 indicates that the ith channel is set to the jth group. Similarly, $t^{k}$(*i*, *j*) = 1 indicates that the ith filter is set to the jth group. The elements of $S^{k}$ and $T^{k}$ are learned during the training process of the CNNs. As shown in Figure 8, the outputs of the four dilated convolutions are aggregated through an element-wise sum operation. Consequently, the size of receptive-field is increased and multi-scale spatial dependencies are considered without resorting to fully connected layers, which would be computationally infeasible. The LPDGC block helps capture the global context in CT images without reducing the resolution of the segmentation map.

#### Feature Attention Module (FAM)

**Figure 9:**
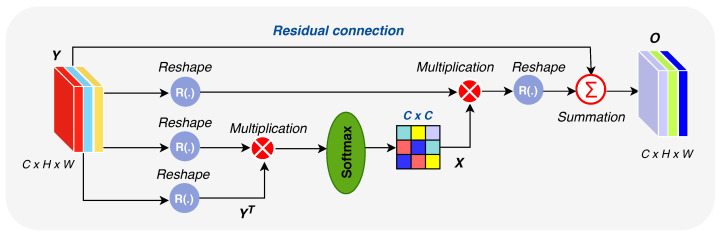
Diagram of our feature attention module (FAM).

Feature attention modules (FAMs) [32] were recently used to encourage CNNs to learn and focus on task-relevant information instead of learning non-useful information (background, non-desired objects, etc.). As one can see in Figure 9, FAM computes the final feature of each channel as a weighted a sum of the features of all channels and original features, which helps boost COVID-19-relevant information and learn semantic dependencies between all feature maps. It should be noted that R(.) refers to reshaping *Y* to $R^{C\times N}$. As shown in Figure 9 (the lower branch), the input feature vector $Y\in R^{C\times H\times W}$ is multiplied by its transposed $Y^{T}$, and the resulting vector is fed into a softmax layer to get the channel attention map $X\in R^{C\times C}$. The final output *O* is obtained as follows:

(4) $$O=\beta \sum_{i=1}^{C}x_{j}i_{Yi}⊕Y_{j}$$

where β is the weight factor, and ⊕ refers to element-wise sum operation.

### 2.2. Decoder Network

**Figure 10:**
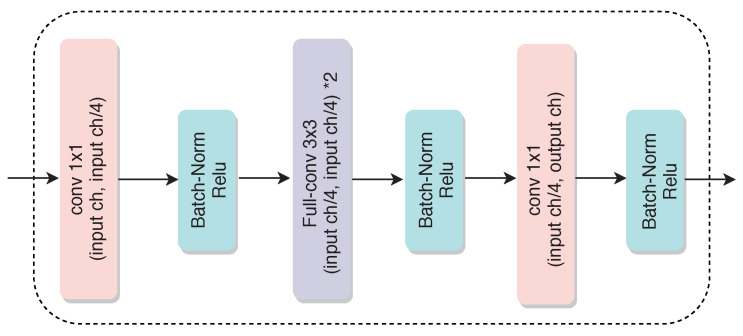
Illustration of the decoder network.

Figure 10 shows the architecture of the decoder network, which consists of four main decoding blocks. The fully-convolutional approach proposed in [15] is employed. This first convolutional layer decreases the overall computational cost by adding a 1×1 kernel. Upsampling layers with a factor *2 are used to upsample the resulting features, and then they are added to the features coming from the corresponding encoder layers via skip connections (as shown in Figure 2). A threshold of 0.5 is employed to convert the output to binary masks. The segmented binary mask has the same size as that of the input image.

### 2.3. Architecture of LungINFseg

Table 1 describes the architecture of LungINFseg. We present the layers of the encoder and decoder, including the input and output feature maps with the number of strides, kernel size, and padding. It should be noted that the input of each encoder block is bypassed to the output of its identical decoder block to recover the spatial feature information [33].

### 2.4. Loss Functions

In this work, we used block-wise loss (BWL) and total loss (TL) functions. In the case of the BWL function, we used dice loss function to compare the features extracted by each RFA block. The BWL function can be formulated as follows:

(5) $$L^{\mathrm{BWL}}\left(y,\hat{y}\right)=\sum_{i=1}^{N}\sum_{c=1}^{H}1-dice\left(y_{ci},{\hat{y}}_{ci}\right),$$

where *N* is the number of RFA blocks, *H* is the number of channels generated by RFA block *i*, y represents the ground-truth, $y_{ci}$ represents the corresponding feature maps, $\hat{y}$ is the predicted mask, ${\hat{y}}_{ci}$ represents the feature maps generated by the RFA blocks, and dice is the Dice coefficient that can be expressed as follows:

(6) $$L^{\mathrm{dice}}\left(y,\hat{y}\right)=1-dice\left(y,\hat{y}\right)=1-\frac{2|y|.|\hat{y}|}{{\left|y\right|}^{2}+{\left|\hat{y}\right|}^{2}},$$

Regarding the TL function, we calculated the loss of the whole network as follows:

(7) $$L^{\mathrm{TL}}\left(y,\hat{y}\right)=-(ylog\left(\hat{y}\right)+\left(1-y\right)log\left(1-\hat{y}\right))+L^{\mathrm{dice}}\left(y,\hat{y}\right)$$

The overall loss (OL) function used for training the proposed model is formulated as:

(8) $$L^{\mathrm{OL}}=L^{\mathrm{BWL}}\left(y,\hat{y}\right)+L^{\mathrm{TL}}\left(y,\hat{y}\right)$$

### 2.5. Evaluation Metrics

To assess the performances of the segmentation models, five evaluation metrics were used: accuracy (ACC), dice coefficient (DSC), intersection over union (IoU), sensitivity (SEN), and specificity (SPE). The formulations of these metrics are given in Table 2.

## 3. Experimental Results and Discussion

In this section, the experimental details, the ablation study, the results of the proposed model, and the comparisons with state-of-the-art models are provided.

### 3.1. Experimental Details

#### 3.1.1. COVID-19 Lung CT Dataset

To evaluate the efficacy of the proposed model, we employed the publicly available dataset provided in [34], which contains 20 labeled COVID-19 CT scans (1800 + annotated slices). This dataset can be found at https://zenodo.org/record/3757476#.X-T7P3VKhhE. Left lung, right lung, and infections were marked by two radiologists and confirmed by an experienced radiologist. The dataset was divided (patient-wise) into three subsets: 70% for training, 10% for validation, and 20% for testing.

#### 3.1.2. Data Augmentation and Parameter Setting

Data augmentation techniques were applied during the training phase to improve the performance of the model and robustness. To augment the CT dataset, we conducted the following procedures: (1) we scaled the images by varying the scaling variable from 0.5 to 2.0 with a step size of 0.25, (2) we employed the gamma correction on the CT slices by changing the gamma scaling constant from 0.5 to 1.5 with a step size of 0.5, and (3) we performed the flipping operations (horizontally and vertically) with 0.5 and rotated them with various angles, such as 15.

Besides, lung CT images were resized to 256×256 pixels. Finally, we normalized each wavelet to [0, 1] to get the input of its corresponding binary segmentation network. It should be noted that LungINFseg processes each CT volume slice by slice. The hyperparameters of the model were empirically tuned. We examined numerous optimizers, such as SGD, AdaGrad, Adadelta, RMSProp, and Adam, while changing the learning rate; we obtained the best outcomes with the Adam optimizer with β1 = 0.5, β2 = 0.999, and learning rate = 0.0002 with a batch size of four. We trained all segmentation models from scratch for 100 epochs. The experiments were carried out on an NVIDIA GeForce GTX 1070Ti with 8 GB of video RAM. The operating system was Ubuntu 18.04 using a 3.4 GHz Intel Core-i7 with 16 GB of RAM. The main required packages involve Python 3.6, CUDA 9.1, cuDNN 7.0, and PyTorch 0.4.1. To reproduce the results, the source code of the proposed model is publicly available at https://github.com/vivek231/LungINFseg.

### 3.2. Ablation Study

To demonstrate the impact of each block on the performance of the proposed model, an ablation study was done. We firstly trained a baseline model without appending the discrete wavelet transform (DWT), learnable parallel dilated group convolutional (LPDGC) block, or feature attention module (FAM). Next, we added DWT to the baseline model (baseline + DWT). Besides, the LPDGC block was added separately to the baseline model (baseline + LPDGC). Apart from this, we also added FAM to each encoding layer of the baseline model (baseline + FAM). Several configurations were investigated, such as baseline + DWT + LPDGC and baseline + DWT + FAM. Finally, we studied the performance of the proposed model with and without data augmentation.

Table 3 presents the results of different configurations of the examined models. The baseline model yielded DSC and IoU scores of 75.56% and 61.96% respectively. From this initial check, there is a possibility of improvement in model performance. Alternatively to adding a gray-scale channel from lung CT images, we substituted the encoder input by adding DWT to baseline (baseline + DWT); note that DWT produces four channels that carry multi-scale (multi-bands) features. Baseline + DWT achieved gains of 1% and 1.5% in DSC and IoU scores, respectively, when compared to the baseline model.

**Figure 11:**
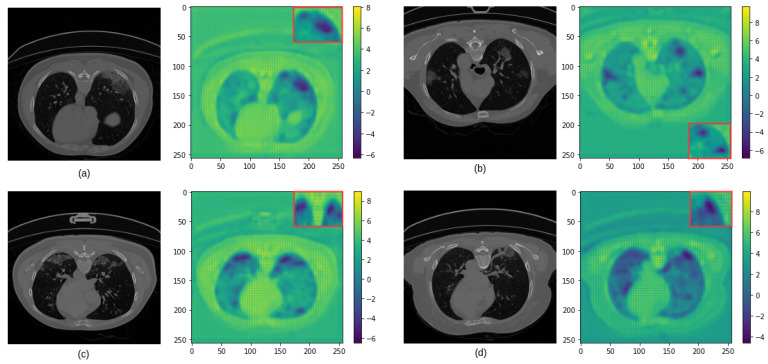
The role of LPDGC in capturing small COVID-19 lung infections from CT images (represented in dark blue). (**a**–**d**) present examples of COVID-19 infection in CT images (**left**) and the corresponding Heatmaps (**right**). Here, the red box presents a zoom-in visualization of the infected region.

Furthermore, the LPDGC block was added to the baseline model to expand the receptive field with varying dilation rates, with the various sizes of kernels, allowing dense feature extraction in the encoder. Figure 11 reveals that the LPDGC block can help capture some small infected regions from lung CT images. Baseline + LPDGC yielded clear improvements of 1.5% and 2% in the DSC and IoU scores respectively, when compared to the baseline model. Baseline + FAM yielded an enhancement in all evaluation matrices, as it achieved 1.5–2% improvements in DSC, IoU, and SEN scores, meaning the FAM block helps to improve the feature discriminability between a given COVID-19-infected region and neighboring healthy pixels.

Based on the significant enrichment of each block, DWT and LPDGC blocks were combined with the baseline model, which led to improvements of more than 2% in DSC, IoU, and SEN scores, and a decrement in the standard deviation by 1%. Besides, we added DWT and FAM to the baseline model, which allowed us to create descriptive features to highlight the infected region in poor contrast or fuzzy-boundary CT images. The experiments revealed that this configuration yields small increases on the evaluation metrics compared to previous results.

Using the proposed LungINFseg model, we experimented with varying configurations with and without applying data augmentation during the training procedure. Without implementing data augmentation (w/o augmentation), LungINFseg obtained encouraging 3% improvements in DSC and IoU scores when compared to the baseline model. Finally, we utilized data augmentation (with augmentation) with LungINFseg. The performance of LungINFseg was improved by 5–6% in DSC, IoU, and SEN scores. The standard deviation of LungINFseg was reduced from ±0.12 to ±0.10. These effects reveal that LungINFseg can present more precise and robust segmentation compared to the baseline model.

### 3.3. Analysis of the Performance of the Proposed Model

**Figure 12:**
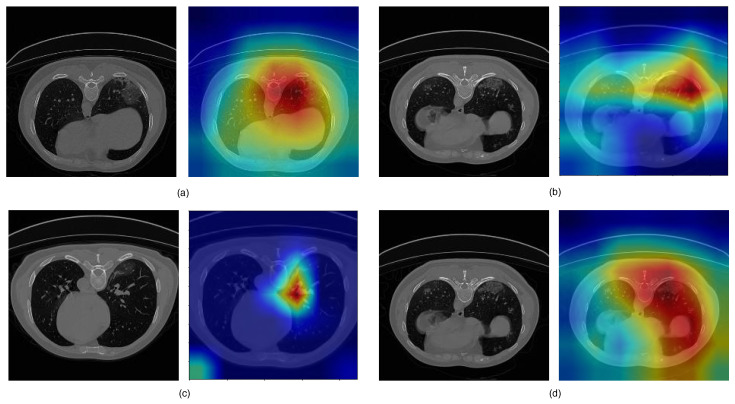
Different examples of channel attention maps (CAMs) obtained from the LungINFseg. (**a**–**d**) present examples of COVID-19 infection in CT images (**left**) and the corresponding CAMs (**right**).

Figure 12 presents four samples of channel attention maps (CAMs) of lung CT COVID-19 infection images. The CAMs shown in the figure were obtained from the different encoding layers of LungINFseg. The red refers to a higher probability of the presence of infection in the lung, while the blue represents a lower probability of the existence of an infection region. As shown, LungINFseg helps highlight infected regions while supplying less attention to neighboring pixels.

Table 4 presents the effectiveness of input image resolution on the performance of the proposed model (512×512, 384×384, 256×256, and 128×128). With the image resolution 512×512, a 16×16 feature map was produced at the final encoding layer, which extracts infected region features from CT images. However, the use of higher resolution images keeps some artifacts at the segmented masks, leading to DSC and IoU scores of 78.74% and 66.48%, respectively. With the image resolution 384×384, a 12×12 feature map was produced at the final encoding layer. This image resolution did not contribute to advancing the results. In turn, the image size of 256×256 yielded an 8×8 feature map at the final encoding layer. This feature map preserves infected-area-associated features and discards the irrelevant ones. Lastly, we examined an input size of 128×128; we found that it yielded unclear boundary results in the segmented masks.

Table 5 presents the performance of the proposed model with different combinations of loss functions: BCE (i.e., TL without dice loss–Equation (7)), BCE + IoU-binary, BCE + SSIM [35], BCE + dice loss (i.e., TL–Equation (7)), and TL + BWL (OL–Equation (8)). As shown, all loss functions achieved a dice score higher than 73%. The IoU-binary and SSIM loss function did not achieve a promising IoU score (60.22% and 58.18%, respectively). The convergence of these two loss functions is not significant enough to achieve optimal performance. The best dice and IoU scores are achieved with OL, and therefore it has been utilized with the proposed model.

### 3.4. Comparisons with the State-of-the-Art

To segment the COVID-19 infection from lung CT images, LungINFseg is compared with the state-of-the-art segmentation models, such as FCN [15], UNet [16], SegNet [17], FSSNet [18], SQNet [19], ContextNet [20], EDANet [21], CGNet [22], ERFNet [23], ESNet [24], DABNet [25], Inf-Net [12], and MIScnn [26] models. All these models are assessed both quantitatively and qualitatively. For the quantitative study, segmentation accuracy is computed using the ACC, DSC, IoU, SEN, and SPE. For a fair comparison, the trainable parameters of the individual evaluated model are also provided. In turn, for the qualitative study, prediction with their corresponding ground truth binary masks are compared visually.

As shown in Table 6, LungINFseg achieved the highest DSC of 80.34% and the highest IoU of 68.77%. As for IoU, LungINFseg was significantly improved from 60.87% to 68.77% on the test set compared to the best competitor, FCN. Besides, the second-best competitor DABNet obtained 74.03% and 60.03% DSC and IoU scores respectively; its depth-wise asymmetric bottleneck module generates a sufficient receptive field and densely utilizes the contextual information. In comparison with the results of the very popular baseline biomedical segmentation model called UNet, LungINFseg exceeds it by more than 10% in both DSC and IoU scores. Additionally, SegNet achieved acutely poor outcomes in all matrices considering it is inefficient to segment accurately by producing many numbers of false positives. In turn, FSSNet has very few parameters (0.17 M); it yielded a 67.89% DSC score and failed to restore the infected region’s spatial information at the output level. In the same manner, SQNet did not perform properly, but compared to LungINFseg yielded more than 22% improvements in DSC and IoU scores.

Besides, the ContextNet creates a poor result, as it fails to retain the global context information efficiently, and LungINFseg shows 10% gains in DSC and IoU scores. Nevertheless, EDANet has performed slightly better—70.32%—DSC score because of its dilated convolution and dense connectivity aid to attain the greater result. Further, CGNet has shown some advancement due to its learning capability of the joint features of both local features and neighboring context. However, it misses getting much more global information to form effective segmentation. This model yields 71.21% DSC and 56.83% IoU scores, but LungINFseg has promising increases of 9% and 12% in both DSC and IoU scores respectively.

Two models, ERFNet and ESNet, employed residual 1-D factorized convolutions in encoding layers to extract important features and support to decrease the computation cost (2.06 M and 1.65 M of ERFNet and ESNet respectively). Extracted features do not significantly present a contribution to increasing the feature learnability; and LungINFseg achieved better results of around 7%, 9%, and 12% in DSC, IoU, and SEN scores respectively. Moreover, we have compared the results of our model with the Inf-seg model [12]. As one can see, LungINFseg yields a 12%, significant improvement in the DSC score.

Additionally, we have trained MIScnn [26] from scratch and then compared it with LungINFseg, finding that our model outperforms the results of MIScnn in terms of all evaluation metrics. Unlike the models mentioned-above, LungINFseg has a great generalization ability to segment the infection areas from lung CT images, thanks to RFA and DWT modules that can enlarge the receptive field of the segmentation models and increase the learning ability of the model without information loss.

**Figure 13:**
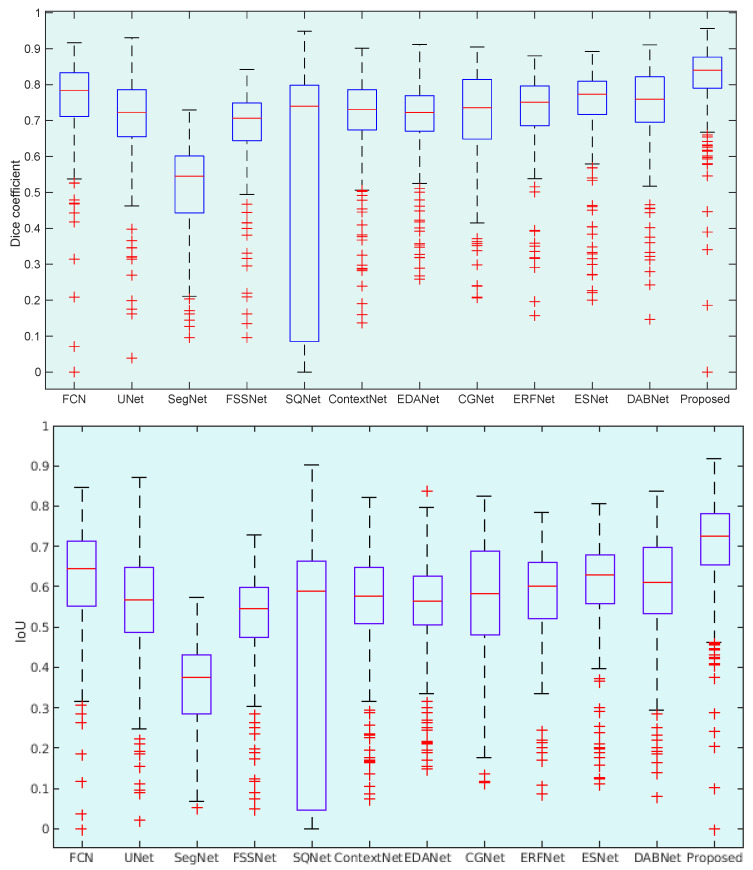
Boxplots of Dice coefficient and Intersection over Union (IoU) scores for all test samples of lung CT infection. Different boxes indicate the score ranges of several methods; the red line inside each box represents the median value; box limits include interquartile ranges Q2 and Q3 (from 25% to 75% of samples); upper and lower whiskers are computed as 1.5 times the distance of upper and lower limits of the box; and all values outside the whiskers are considered as outliers, which are marked with the (+) symbol.

To demonstrate the ability of LungINFseg, we present illustrative statistics of Dice and IoU scores. In Figure 13, we show the boxplots of the Dice and IoU scores of the proposed model, FCN, UNet, SegNet, FSSNet, SQNet SQNet, ContextNet, EDANet, CGNet, ERFNet, ESNet, and DABNet. As shown in Figure 13, among the tested models, the proposed model has the highest mean DSC and IoU scores and the smallest standard deviation with few outliers. In turn, other rest models have represented multiple outliers with low mean and high standard deviation compared to LungINFseg.

**Figure 14:**
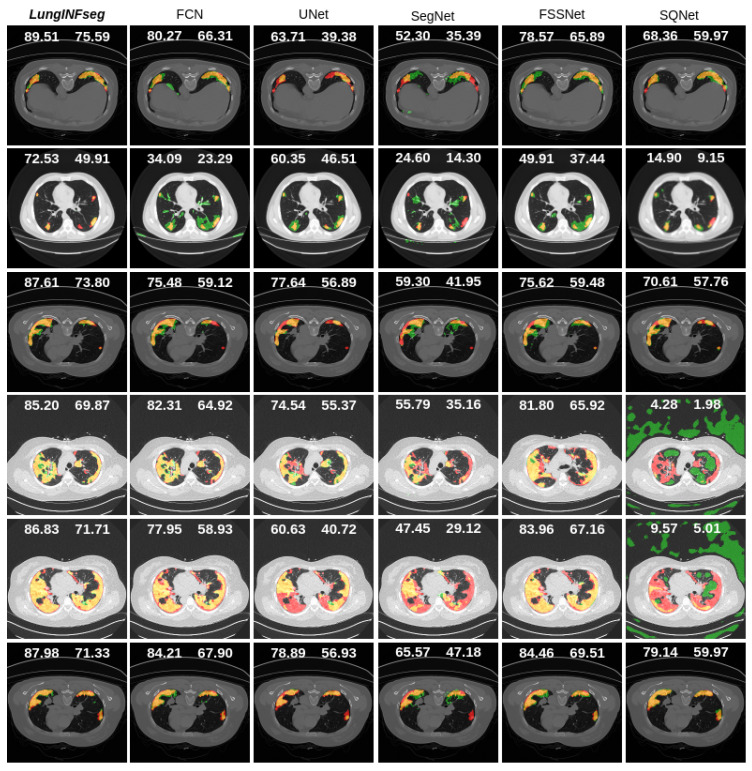
Qualitative comparison of the segmentation results of LungINFseg and five state-of-the-art segmentation methods (left to right: LungINFseg-SQNet). Here, left and right side numbers on each example refer to dice and IoU scores, respectively. The colors used to represent the segmentation results are as follows: TP (orange), FP (green), FN (red), and TN (black).

**Figure 15:**
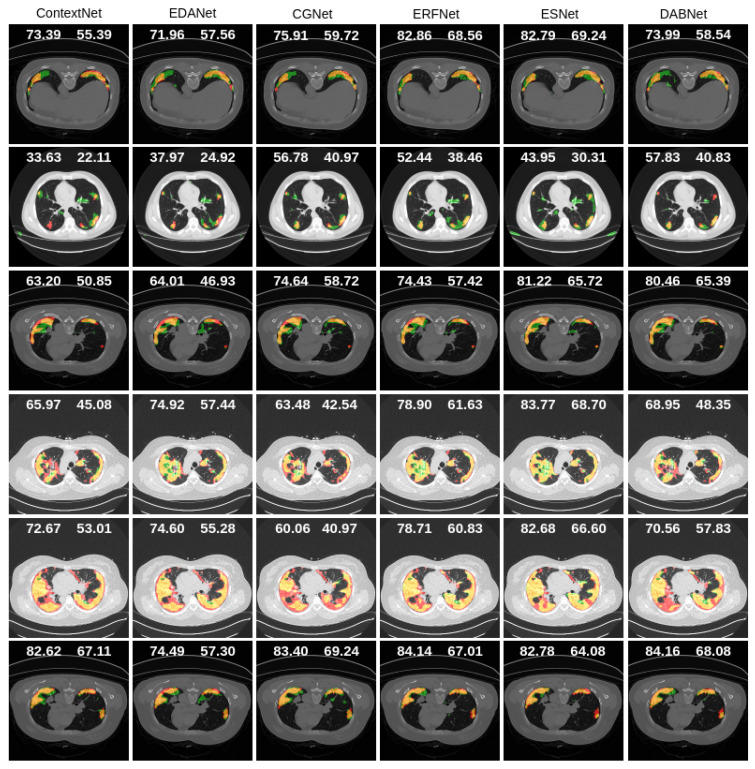
Qualitative comparison of the segmentation results of LungINFseg and six state-of-the-art segmentation methods (left to right: ContextNet–DABNet). Here, left and right side numbers on each example refer to dice and IoU scores, respectively. The colors are used to represent the segmentation results as follows: TP (orange), FP (green), FN (red), and TN (black).

**Figure 16:**
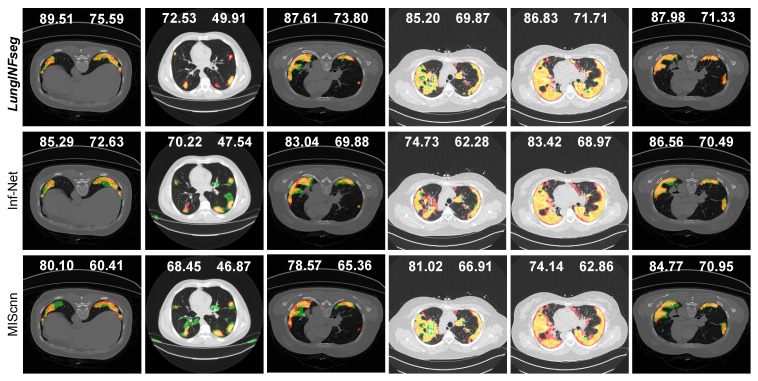
Qualitative comparison of the segmentation results of the LungINFseg, Inf-Net, and MIScnn. Here, left and right side numbers on each example refer to dice and IoU scores, respectively. The colors are used to represent the segmentation results as follows: TP (orange), FP (green), FN (red), and TN (black).

Figure 14, Figure 15 and Figure 16 present qualitative segmentation outcomes of COVID-19 infection from a lung CT image that incorporates a variety of challenging circumstances: illumination variations, irregular boundary, and shape of the infected areas. We have shown six samples as examples along with ground truth and the mask generated by each state-of-the-art method compared to LungINFseg.

In Figure 14 and Figure 15, for example, first, third, and sixth rows have confirmed single infected regions in both sides of the lung and a very small area covered on the left side of the lung. We clearly recognized that LungINFseg is capable of correctly segmenting both side’s lungs, and the other model has carried larger false positives to do an inaccurate segmentation. Furthermore, for example, fourth and fifth rows have a widespread infection on both sides of the lung. In order to provide promising segmentation, LungINFseg segmented quite properly; despite that, FCN has created an acceptable segmentation. However, UNet, FSSNet, ContextNet, EDANet, CGNet, and ESNet generated very poor predictions due to lacking details contained in the low-level context information. Moreover, second row predictions present single small areas of infection, where LungINFseg shows its promising ability to properly segment infected areas, and the other compared methods produced larger false positive predictions. Figure 16 presents a quantitative comparison of the segmentation results of LungINFseg, Inf-Net, and MIScnn models. As one can see in the examples of the second, third, fourth and sixth columns, LungINFseg can accurately segment COVID-19 infection and has fewer FP compared to the Inf-Net and MIScnn models. The proposed model is especially useful for the segmentation of an infection with an indefinite boundary and small targets.

## 4. Conclusions

In this article, we have introduced an efficient deep learning-based LungINFseg model to segment the COVID-19 infection in lung CT images. Specifically, we have proposed the RFA module that can enlarge the receptive field of the segmentation models and increase the learning ability of the model without any information loss. We conducted extensive experiments that used 1800+ annotated CT slices to build and test LungINFseg. Further, we compared LungINFseg with 13 state-of-the-art deep learning-based segmentation methods to demonstrate its effectiveness. LungINFseg achieved a dice score of 80.34% and an IoU score of 68.77%, which are higher than the ones of the other 13 segmentation methods. Our experiments revealed that the RFA module, which allows enlarging receptive fields and encourages learning contextual information and COVID-19 infection-related features, yields accurate segmentation results. We found that LungINFseg can segment infected regions in CT images accurately and may have promising clinical potential. In future work, we will integrate our proposed model with a fully automated CAD system for making an accurate predictions for the severity of COVID-19. Besides, we will apply LungINFseg to different medical image segmentation problems, such as lung lobe segmentation, skin lesion segmentation, and breast tumor segmentation in ultrasound images.

## Figures and Tables

**Table 1:** Architecture details of LungINFseg. Skip connection is used to connects the encoder layers with the corresponding decoder layers to preserve the spatial information.

	Layer	Type	Input Feature Size	Stride	Kernel Size	Padding	Output Feature Size
**ENCODER**	1	Initial block with DWT	n × 1 × 256 × 256	1	7	3	n × 64 × 128 × 128
	2	RFA Block 1	n × 64 × 128 × 128	1	3	1	n × 64 × 64 × 64
	3	RFA Block 2	n × 64 × 64 × 64	2	3	1	n × 128 × 32 × 32
	4	RFA Block 3	n × 128 × 32 × 32	2	3	1	n × 256 × 16 × 16
	5	RFA Block 4	n × 256 × 16 × 16	2	3	1	n × 512 × 8 × 8
**DECODER**	6	Block 1	n × 512 × 8 × 8	2	3	1	n × 256 × 16 × 16
	7	Block 2	n × 256 × 16 × 16	2	3	1	n × 128 × 32 × 32
	8	Block 3	n × 128 × 32 × 32	2	3	1	n × 64 × 64 × 64
	9	Block 4	n × 64 × 64 × 64	1	3	1	n × 64 × 64 × 64
	9	ConvTranspose	n × 64 × 64 × 64	2	3	1	n × 32 × 128 × 128
	9	Convolution	n × 32 × 128 × 128	1	3	1	n × 32 × 128 × 128
	10	ConvTranspose (Output)	n × 32 × 128 × 128	2	2	0	n × classes (1) × 256 × 256

**Table 2:** Metric used to evaluate the segmentation methods.

Metric	Formula
Accuracy (ACC)	(TP + TN) / (TP + TN + FP + FN)
Dice coefficient (DSC)	2.TP / (2.TP + FP + FN)
Intersection over Union (IoU)	TP / (TP + FP + FN)
Sensitivity (SEN)	TP/(TP + FN)
Specificity (SPE)	TN / (TN + FP)

TP = True Positives; TN = True Negatives; FP = False Positives, FN = False Negatives.

**Table 3:** Investigating the performance of different configurations of the proposed method (mean ± standard deviation). Best results are in bold.

Model	ACC (%)	DSC (%)	IoU (%)	SEN (%)	SPE (%)
Baseline	0.9844±0.0098	0.7556±0.1170	0.6195±0.1268	0.7762±0.1479	0.9920±0.0041
Baseline + DWT	0.9847±0.0092	0.7641±0.1134	0.6361±0.1202	0.7888±0.1446	0.9927±0.0040
Baseline + LPDGC	0.9859±0.0088	0.7696±0.1117	0.6415±0.1127	0.7892±0.1426	0.9934±0.0036
Baseline + FAM	0.9860±0.0080	0.7703±0.1092	0.6389±0.1164	0.7974±0.1391	0.9939±0.0032
Baseline + DWT + LPDGC	0.9865±0.0074	0.7742±0.1070	0.6420±0.1151	0.8062±0.1403	0.9942±0.0030
Baseline + DWT + FAM	0.9867±0.0062	0.7785±0.1058	0.6510±0.1142	0.8148±0.1395	0.9944±0.0028
LungINFseg (w/o augmentation)	0.9874±0.0057	0.7853±0.1050	0.6522±0.1136	0.8229±0.1374	0.9948±0.0025
**LungINFseg (with augmentation)**	0.9892±0.0054	0.8034±0.1021	0.6877±0.1095	0.8310±0.1323	0.9952±0.0022

**Table 4:** The performance of the LungINFseg with different image resolutions on the test set (mean ± standard deviation). Best results are in bold.

Input Size	ACC	DSC	IoU	SEN	SPE	Feature Map Size
512×512	0.9856±0.0090	0.7874±0.1098	0.6648±0.1167	0.8007±0.1386	0.9938±0.0032	16×16
384×384	0.9841±0.0095	0.7623±0.1143	0.6350±0.1233	0.7862±0.1456	0.9830±0.0037	12×12
256×256	0.9892±0.0054	0.8034±0.1021	0.6877±0.1095	0.8310±0.1323	0.9952±0.0022	8×8
128×128	0.9681±0.0126	0.7025±0.1576	0.5864±0.1940	0.7111±0.1611	0.9810±0.0052	4×4

**Table 5:** The evaluation of the LungINFseg with different loss functions on the test set (mean ± standard deviation). Best results are in bold.

Loss Function	ACC (%)	DSC (%)	IoU (%)	SEN (%)	SPE (%)
BCE	0.9832±0.0087	0.7705±0.1187	0.6441±0.1258	0.7907±0.1594	0.9910±0.0049
BCE + IoU-binary	0.9717±0.0095	0.7450±0.1297	0.6022±0.1377	0.7653±0.1580	0.9862±0.0060
BCE + SSIM	0.9630±0.0099	0.7302±0.1465	0.5818±0.1536	0.7594±0.1748	0.9779±0.0090
TL	0.9865±0.0075	0.7862±0.1067	0.6523±0.1146	0.8004±0.1401	0.9927±0.0031
**LungINFseg (OL)**	0.9892±0.0054	0.8034±0.1021	0.6877±0.1095	0.8310±0.1323	0.9952±0.0022

**Table 6:** Comparing the proposed model with 13 state-of-the-art baseline segmentation methods on the test set (mean ± standard deviation). Best results are in bold. Dashes-indicate that the information is not reported in the cited references.

Model	ACC (%)	DSC (%)	IoU (%)	SEN (%)	SPE (%)	Parameters (M)
FCN	0.9885±0.0095	0.7422±0.1182	0.6087±0.1290	0.7745±0.1482	0.9950±0.0029	135.53
UNet	0.9861±0.0116	0.7039±0.1298	0.5565±0.1366	0.7057±0.1437	0.9963±0.0038	14.78
SegNet	0.9741±0.0107	0.5095±0.1307	0.3515±0.1508	0.6900±0.1902	0.9851±0.0064	29.44
FSSNet	0.9867±0.0118	0.6789±0.1198	0.5246±0.1302	0.6257±0.1559	0.9969±0.0034	0.17
SQNet	0.9555±0.0632	0.5451±0.3375	0.4424±0.2927	0.5624±0.3446	0.9699±0.0463	16.23
ContextNet	0.9872±0.0099	0.7081±0.1299	0.5617±0.1366	0.7353±0.1725	0.9950±0.0041	0.87
EDANet	0.9861±0.0095	0.7032±0.1175	0.5536±0.1359	0.7718±0.1590	0.9933±0.0055	0.68
CGNet	0.9862±0.0124	0.7121±0.1339	0.5683±0.1491	0.7624±0.2083	0.9946±0.0046	0.49
ERFNet	0.9882±0.0111	0.7260±0.1120	0.5801±0.1294	0.6913±0.1358	0.9973±0.0032	2.06
ESNet	0.9897±0.0088	0.7381±0.1251	0.5979±0.1328	0.7031±0.1361	0.9970±0.0034	1.65
DABNet	0.9879±0.0105	0.7403±0.1199	0.6003±0.1339	0.7594±0.1628	0.9951±0.0043	0.75
Inf-Net [12]	−	0.6820	−	0.6920	0.9430	33.12
MIScnn [26]	0.9880±0.0102	0.7365±0.1185	0.6074±0.1299	0.7719±0.1491	0.9872±0.0030	13.60
**LungINFseg**	0.9892±0.0074	0.8034±0.1021	0.6877±0.1095	0.8310±0.1323	0.9952±0.0022	11.54

## Data Availability

The dataset used in this study can be found at https://zenodo.org/record/3757476#.X-T7P3VKhhE.
